# Uterine perivascular epithelioid tumors (PEComas) with lung metastasis showed good responses to mTOR and VEGFR inhibitors: A case report

**DOI:** 10.3389/fonc.2022.797275

**Published:** 2022-07-28

**Authors:** Chengxu Sui, Jie Wu, Dan Mei, Evenki Pan, Peng Yang, Tingting Wu, Yutong Ma, Qiuxiang Ou, Lei Song

**Affiliations:** ^1^ Department of Intervention Therapy, The Second Hospital of Dalian Medical University, Dalian, China; ^2^ Geneseeq Research Institute, Nanjing Geneseeq Technology Inc., Nanjing, China

**Keywords:** uterine PEComa, everolimus, apatinib, *TSC2*, lung metastasis

## Abstract

Perivascular epithelioid cell tumors (PEComas) are extremely rare mesenchymal neoplasms for which the uterus is the most common site. The prognosis of malignant PEComa is poor as it is characterized by resistance to classical chemotherapies. Both mTOR inhibitors and VEGFR inhibitors exhibited clinical utility in treating malignant PEComas, but the combination of these two regimens has rarely been reported. In the present case, a uterine PEComa patient developed lung and bone metastases after the failure of chemotherapies and derived benefit from the combination regimen of an mTOR inhibitor (everolimus) and a VEGFR inhibitor (apatinib), achieving a 15-month progression-free survival. Targeted NGS revealed *TP53* and *TSC2* mutations in the patient’s primary uterine tumors and plasma ctDNA at disease progression. Plasma ctDNA clearance was consistent with a radiologic partial response determined by RECIST 1.1 and a reduction of neuron-specific enolase (NSE) and cancer antigen 125 (CA125) levels. Thus, we provided clinical evidence supporting the administration of combined therapy of mTOR and VEGFR inhibitors to metastatic uterine PEComa patients and highlighted the application of serial plasma ctDNA profiling for dynamic disease monitoring.

## Introduction

Perivascular epithelioid cell tumors (PEComas) are rare mesenchymal neoplasms containing epithelioid cells with a perivascular distribution and are characterized by immunophenotypic features of smooth muscle and melanocytic differentiation (1). The main members of PEComas include angiomyolipoma (AML) and pulmonary lymphangioleiomyomatosis (LAM), which are often characterized by a benign clinical course and are observed at high frequencies in patients with tuberous sclerosis complex (TSC). While the presence of aggressive PEComas is usually accompanied by locally invasive recurrences or distant metastases, a gynecologic-specific algorithm has been proposed to classify malignant PEComas, including the following atypical features: size ≥5 cm, high-grade atypia, mitoses >1/50 high-power fields (HPF), necrosis, and vascular invasion (2).

Studies of the genetic changes in PEComas revealed a high incidence of *TSC1* or *TSC2* alterations, which constitutively activated the mTOR pathway and promoted translational initiation and cell growth (3, 4). mTOR inhibitors, including sirolimus, everolimus, and temsirolimus, were used in malignant PEComa patients with clinical benefits (5–7). At present, multiple clinical trials are actively investigating the clinical benefit of mTOR inhibitors including everolimus and sirolimus in advanced solid tumors with inactivating *TSC1* or *TSC2* mutation (NCT02352844, NCT02201212, and NCT05103358). In addition, *TFE3* rearrangements were reported in some cases with wild-type *TSC1/2*, indicating that alternative pathways of tumorigenesis exist and that alternative treatment strategies are needed (8, 9). Other gene alterations such as *ATRX* mutations, *RB1* deletions, and the amplification of *FGFR3*, *NTRK1*, and *ERBB3* were also detected by targeted massively parallel sequencing (10). Except a clinical trial evaluating the benefit of erdafitinib in patients carrying *FGFR3* gene amplification, more effort needs to be made to develop effective therapy targeting other mentioned genomic aberrations.

The management of malignant PEComas is challenging, and systemic chemotherapy has shown little efficacy in retrospective studies (11–13). The response to VEGFR inhibitors has also been suboptimal, with very low objective response rates (ORR) (8.3%) (11). Data on the combination of mTOR and VEGFR inhibitors for the treatment of malignant PEComas are limited, but one case reported a remarkable response for the treatment of a uterine PEComa patient with kidney and lung metastases using sirolimus and sorafenib (14). Herein, we present a malignant uterine PEComa patient who developed lung and bone metastases after the failure of chemotherapy but responded well to the combined therapy of mTOR and VEGFR inhibitors, with a 15-month progression-free survival (PFS).

## Case presentation

**Figure 1:**
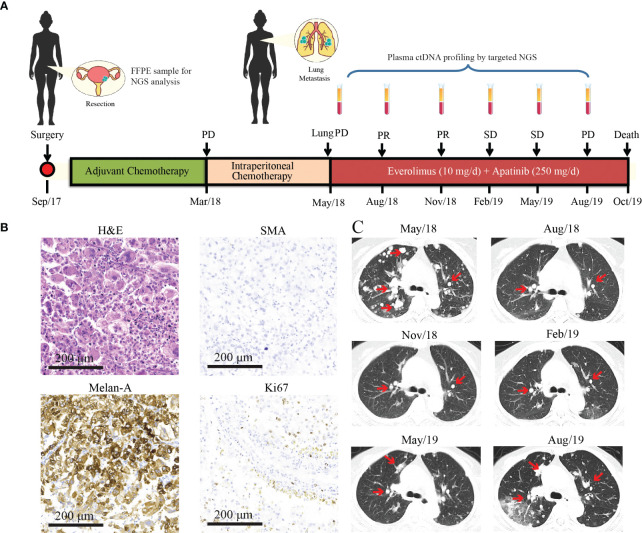
Treatment history and clinical information of the presented case. **(A)** The medical history of the presented case is shown with information about treatment timeline, response evaluation, and sample collection timepoints. During the combination treatment with everolimus and apatinib, plasma ctDNA sequencing was performed every 3 months along with treatment response evaluation as indicated by the arrowheads. **(B)** H&E staining and immunohistochemical (IHC) examinations (×200) of the primary uterine PEComa which was negative for the SMA marker and positive for Melan-A (90%). The Ki67 index is 20%. **(C)** CT images of lung metastases during everolimus and apatinib treatment. Lesions are indicated by the red arrows. PR, partial response; SD, stable disease; PD, progressive disease; NGS, next-generation sequencing; PEComa, perivascular epithelioid cell tumors; ctDNA, circulating tumor DNA; FFPE, formalin-fixed, paraffin-embedded; H&E, hematoxylin and eosin; SMA, smooth muscle actin.

**Figure 2:**
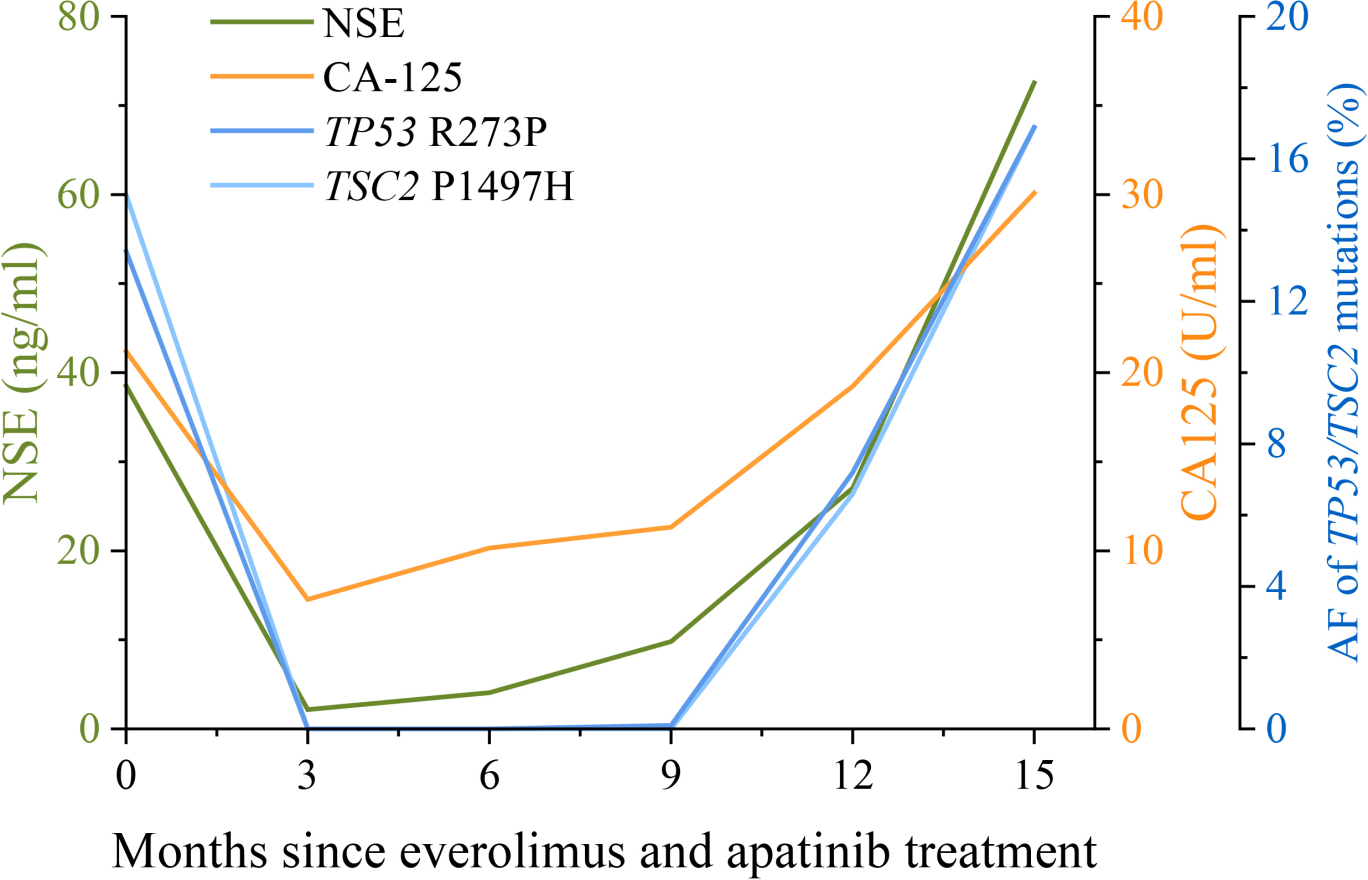
Changes in NSE and CA125 levels, and the allele frequencies (AFs) of *TP53* and *TSC2* mutations during apatinib and everolimus treatment. The levels of the lung cancer biomarkers NSE (neuron-specific enolase) and CA125 (cancer antigen 125) in serum examined every 3 months are shown by the green and orange lines, respectively. Plasma ctDNA sequencing was also performed every 3 months during apatinib and everolimus treatment. The AFs of *TP53* R273P (blue) and *TSC2* P1497H (light blue) mutations are shown by the dark blue and light blue lines, respectively. The units of NSE, CA125, and AF were indicated by the different y-axes.

A 47-year-old woman without a genetic family history or past diseases presented with lower abdominal pain in September 2017. The color Doppler ultrasound revealed a mass in the right side of the uterus, which was surgically removed (**Figure 1AM**). Immunohistochemistry (IHC) examinations of the resected tumor tissues were positive for melanoma antigen (Melan-A, 90%) and negative for human melanoma black (HMB45), smooth muscle actin (SMA), S-100, desmin, Myo-D1, synaptophysin (Syn), and creatine kinase (CK). Ki67 labeling in the tumor cells was 20% (**Figure 1B**). Necrosis and vascular invasion were observed. Based on the histological and IHC results, the patient was diagnosed with stage Ib malignant uterine PEComa, without metastasis. Four cycles of postoperative adjuvant chemotherapy with epirubicin (90 mg on d1) and cyclophosphamide (2 g on d1–4) were administrated, but obvious side effects with grade 3 myelosuppression were observed. In March 2018, the patient received intraperitoneal chemotherapy with cisplatin (80 mg) and sodium bicarbonate (150 ml), but the disease progressed rapidly with the development of lung and bone metastases within 2 months (**Figure 1C**). The levels of neuron-specific enolase (NSE) and cancer antigen 125 (CA125) were 38.52 and 21.2 U/ml, respectively (**Figure 2**). To identify a more efficient therapeutic strategy, freshly collected plasma and formalin-fixed, paraffin-embedded (FFPE) primary uterine tumor tissues were subjected to targeted next-generation sequencing (NGS) of over 400 cancer-related genes (**Supplementary Methods**). As shown in **Table 1**, the plasma ctDNA exhibited *TP53* (R273P) and *TSC2* (P1497H) mutations, compared to the primary tumor sample, while copy number variants of *ZNF703*, *FGFR3*, *FLT4*, and *RB1* were only detected in the primary tumor. A combined treatment of apatinib (250 mg, once a day) and the mTOR inhibitor everolimus (10 mg, once a day) was administrated in May 2018. Plasma ctDNA sequencing was performed every 3 months until progressive disease (PD), as well as measurements of NSE and CA125. The patient achieved a partial response (PR) after 3 and 6 months of apatinib and everolimus treatment, after which the plasma ctDNA was still tested negative for genomic alterations and the levels of NSE and CA125 were dramatically decreased (**Figure 2**). Stable disease (SD) was observed in February and May 2019, with the positive detection of ctDNA alterations in plasma samples; however, the allele frequencies (AFs) were relatively low (**Table 1**). Additionally, the levels of NSE and CA125 were slightly but continuously increased after 9 and 12 months of combined treatment (**Figure 2**). After 15 months of apatinib and everolimus treatment, the disease progressed with the detection of high-AF *TP53* and *TSC2* mutations, as well as an acquired *ARID1B* (G169R) mutation (**Table 1**). The NSE and CA125 levels were also dramatically elevated. Grade 1–2 nausea and rash were reported during the combined treatment, and the patient died of a respiratory failure in October 2019.

**Table 1:** The allele frequencies of genetic alterations detected by targeted NGS in the primary PEComa tumor and serial plasma ctDNA.

Gene	Alteration	Primary PEComa (FFPE)	Plasma ctDNA(months since everolimus and apatinib treatment)					
			0	3	6	9	12	15
*TP53*	R273P	30.27%	13.41%	–	–	0.10%	7.20%	16.89%
*TSC2*	P1497H	30.88%	15%	–	–	–	6.62%	16.87%
*ZNF703*	CNV	2.9-fold	–	–	–	–	–	–
*FGFR3*	CNV	2.6-fold	–	–	–	–	–	–
*FLT4*	CNV	2.5-fold	–	–	–	–	–	–
*RB1*	CNV	single‐copy loss	–	–	–	–	–	–
*ATRX*	T1545fs	–	9.10%	–	–	–	5.44%	12.83%
*ARID1B*	G169R	–	–	–	–	–	–	54.83%

FFPE, formalin-fixed, paraffin-embedded; “-”, not detected; CNV, copy number variant; ctDNA, circulating tumor DNA

the allele frequency (AF) of the ctDNA mutation was 0.5%. As the TP53 (R273P) mutation was detected in the primary tissue and the first plasma sample had a high AF, a 0.1% mutation AF is reported.

## Discussion

PEComas are rare, and the metastatic sites of malignant PEComas usually include the gastrointestinal tract, lung, retroperitoneum, uterus, and somatic soft tissues (15, 16). The uterus is the most common site of PEComas, but the uterine PEComa presented in this case was negative for the HMB45 marker, which is extremely rare. HMB45 is considered as the most reliable IHC marker for identifying PEComas, with over 95% exhibiting a positive expression (17). However, this case revealed an HMB45-negative profile, suggesting that the diagnosis of PEComas should be based on histological and IHC examinations.

Radical resection is the primary treatment option for uterine PEComas, as they are typically resistant to radiation and chemotherapy. A retrospective study (11) showed an ORR of 13% for anthracycline-based chemotherapy in advanced PEComa patients whose median PFS was 3.2 months. Similarly, that study (11) also showed that the ORR and median PFS in a gemcitabine-based chemotherapy subgroup were 20% and 3.4 months, respectively. In the current case, neither postoperative adjuvant chemotherapy nor intraperitoneal chemotherapy provided optimal outcomes.

Considering the frequent detection of *TSC1/*2 loss-of-function alterations as causing the activation of the mTOR signaling pathway (18), treatment with mTOR inhibitors exhibited clinical benefits to malignant PEComa patients, which were first reported in 2010 (6). Subsequently, the application of mTOR inhibitors in patients with malignant PEComas was demonstrated in additional studies. The efficacy of mTOR inhibitors was better than that of classical chemotherapies, with an ORR of 41% and a 9-month median PFS (11, 19). In the current case, targeted NGS detected a *TSC2* P1497H mutation in the primary uterine PEComa and the plasma ctDNA collected after the occurrence of metastases. Although the clinical significance of this missense mutation remains unknown, we hypothesized that the *TSC2* P1497H mutation might affect the function of TSC2 and further activate the mTOR signaling pathway as the patient benefited from everolimus treatment. However, additional clinical data are needed to support this single-case observation.

Antiangiogenic VEGFR inhibitors also exhibited clinical responses in PEComa patients, but mainly in stabilizing disease in patients with malignant PEComas (ORR = 8.3%, median PFS = 5.4 months) (11, 20). The combination of the VEGFR inhibitor, sorafenib, with the mTOR inhibitor, sirolimus, led to a complete response in a uterine PEComa case reported in 2016; however, the patient’s molecular features were not discussed in the study (14). In the current case, the combined use of the VEGFR inhibitor, apatinib, and the mTOR inhibitor, everolimus, led to the best PR (PFS = 15 months).

In the present case, we also demonstrated the utility of NGS for treatment decision making and response monitoring. Besides the common *TP53* and *TSC2* mutations, amplification of *ZNF703*, *FLT4*, and *FGFR3* was also detected in the primary uterine tumor. The overexpression of ZNF703 was reported to activate the Akt/mTOR signaling pathway in breast cancer cells (21). The consequence of *ZNF703* amplification in PEComas remains to be determined, but it might also contribute to the response to everolimus in this case. *FGFR3* is a predictive biomarker for use of erdafitinib in patients, but no effective therapies target other mentioned genomic aberrations in the presented case. After the failure of chemotherapy, the plasma ctDNA exhibited an *ATRX* frameshift mutation. Additionally, at the time of progression on the combined therapy (everolimus + apatinib), another *ARID1B* mutation was detected. These acquired mutations may inspire the investigations of the resistance to chemotherapy and mTOR inhibitors in PEComa patients, although no studies have reported an association between these acquired mutations and the specific treatments. The differences in genetic alterations between primary and metastatic samples also suggested tumor evolution, which may assist in changing therapeutic strategies. In addition, we also found that serial ctDNA profiling during treatment could forecast disease progression earlier than CT scanning (22). The increase in the mutational AF of plasma ctDNA was observed prior to image-confirmed progression and also displayed a similar trend as the changes in NSE and CA125 levels. NSE is a reliable tumor marker in several cancers, especially in patients with neuroblastoma or small cell lung cancer (23). Similarly, CA-125 is widely used to identify early signs of ovarian cancer (24). Thus, the changes in NSE and CA-125 levels can also assist with disease monitoring in uterine PEComa patients with lung metastases.

The limitation of the single-case presentation in this study should also be noted. Thus, the efficacy and the side effects of the combined treatment with mTOR and VEGFR inhibitors must be further evaluated in larger cohorts. The missense mutation of *TSC2* (P1497H) in this case might be a potential target of mTOR inhibitors; however, additional preclinical studies and additional clinical evidence are needed.

## Conclusion

In summary, we reported a patient with a rare uterine PEComa who harbored a *TSC2* P1497H mutation and received a combined treatment with apatinib and everolimus after chemotherapy failed. The patient’s metastatic lung lesions were stable for 15 months, and serial plasma ctDNA profiling and profiling using the serum tumor markers, NSE and CA125, facilitated disease monitoring. This case detailed a reliable treatment option for rare uterine PEComas with distant metastases and highlighted the importance of longitudinal ctDNA profiling during treatment.

## Data availability statement

The original contributions presented in the study are included in the article/**Supplementary Material**. Further inquiries can be directed to the corresponding author.

## Ethics statement

This research was approved by the Ethics Committee of The Second Hospital of Dalian Medical University (Approval ID: DMU2021139). Written informed consent to publish the clinical details and images were obtained from the patient.

## Author contributions

All authors contributed to data analysis and drafting or revising of the manuscript. All authors agreed on the journal to which the article is submitted, provided final approval of the manuscript version to be published, and agreed to be accountable for all aspects of the study.

## Conflict of interest

Authors EP, PY, TW, YM, and QO are employed by Nanjing Geneseeq Technology Inc.

The remaining authors declare that the research was conducted in the absence of any commercial or financial relationships that could be construed as a potential conflict of interest.

## Publisher’s note

All claims expressed in this article are solely those of the authors and do not necessarily represent those of their affiliated organizations, or those of the publisher, the editors and the reviewers. Any product that may be evaluated in this article, or claim that may be made by its manufacturer, is not guaranteed or endorsed by the publisher.
