# Supplementary material for: Uterine perivascular epithelioid tumors (PEComas) with lung metastasis showed good responses to mTOR and VEGFR inhibitors: A case report
Source: Front Oncol. 2022 Jul 28;12:797275. doi: 10.3389/fonc.2022.797275 (PMC9366196; doi:10.3389/fonc.2022.797275)
Supplement: Supplementary file 1 [file DataSheet_1.docx]

**Supplementary Methods**

*DNA extraction and targeted enrichment*

Whole blood samples were collected in cell-free DNA BCT tubes (Streck Inc., La Vista, NE, USA). The cellular fraction and plasma were separated by two-step centrifugation at 4°C (1900 g for 10 mins and 16,000 g for 10 mins). The white blood cells were used for genomic DNA extraction (DNeasy Blood & Tissue Kit, Qiagen) as germline control. ctDNA from plasma was purified using the Circulating Nucleic Acid Kit (Qiagen) following the manufacturer’s protocol, while FFPE genomic DNA was purified using the QIAamp DNA FFPE Tissue Kit (Qiagen). All DNA was quantified using the dsDNA HS Assay Kit on a Qubit Fluorometer (Life Technologies). Sequencing libraries were prepared using the KAPA Hyper Prep Kit (KAPA Biosystems), as described previously (Shu et al., 2017). Indexed DNA libraries were pooled together for probe-based hybridization capture of the targeted gene regions covering over 400 cancer-related genes for all solid tumors.

*Sequencing data processing*

Sequencing was performed using the Illumina HiSeq4000 platform, followed by data analysis as previously described (Yang et al., 2018). In brief, sequencing data were analyzed by Trimmomatic (Bolger et al., 2014) to remove low-quality (quality <15) or N bases, and were then mapped to the human reference genome, hg19, using the Burrows-Wheeler Aligner (https://github.com/lh3/bwa/tree/master/bwakit). PCR duplicates were removed by Picard (available at https://broadinstitute.github.io/picard/). The Genome Analysis Toolkit (GATK) (https://software.broadinstitute.org/gatk/) was used to perform local realignments around indels and base quality reassurance. Gene fusions were identified by FACTERA (Newman et al., 2014). Somatic SNPs and indels were analyzed by VarScan2 (Koboldt et al., 2012) and Mutect2, with the mutant allele frequency cutoff at 2% for tissue samples, 0.5% for cfDNA samples, and a minimum of three unique mutant reads. Common SNPs were excluded if they were present in >1% population frequency in the 1000 Genomes Project or the Exome Aggregation Consortium (ExAC) 65,000 exomes database. The resulting mutation list was further filtered by an in-house list of recurrent artifacts based on a normal pool of whole blood samples.

*Cytological and immunohistochemistry (IHC) analysis*

Histological analysis of the primary FFPE uterine PEComa was performed with H&E staining. IHC staining for HMB45 (ZM-0187, Zhongshan Golden Bridge Bio-technology), Melan-A (MAB-0275, Fuzhou Maixin Biotechnologies), smooth muscle actin (SMA, kit-0006, Fuzhou Maixin Biotechnologies) S-100 (RAB-0150, Fuzhou Maixin Biotechnologies), desmin (ZA-0610, Zhongshan Golden Bridge Bio-technology), Myo-D1 (ZA-0585, Zhongshan Golden Bridge Bio-technology), synaptophysin (Syn, ZA-0506, Zhongshan Golden Bridge Bio-technology), and CK (Kit-0009, Fuzhou Maixin Biotechnologies), and Ki67 (ZM-0166, Zhongshan Golden Bridge Bio-technology) were performed according to the manufacturer’s protocol by the Department of Pathology, the Second Hospital of Dalian Medical University.

Bolger, A.M., Lohse, M., and Usadel, B. (2014). Trimmomatic: a flexible trimmer for Illumina sequence data. *Bioinformatics* 30(15)**,** 2114-2120. doi: 10.1093/bioinformatics/btu170.

Koboldt, D.C., Zhang, Q., Larson, D.E., Shen, D., McLellan, M.D., Lin, L., et al. (2012). VarScan 2: somatic mutation and copy number alteration discovery in cancer by exome sequencing. *Genome Res* 22(3)**,** 568-576. doi: 10.1101/gr.129684.111.

Newman, A.M., Bratman, S.V., Stehr, H., Lee, L.J., Liu, C.L., Diehn, M., et al. (2014). FACTERA: a practical method for the discovery of genomic rearrangements at breakpoint resolution. *Bioinformatics* 30(23)**,** 3390-3393. doi: 10.1093/bioinformatics/btu549.

Shu, Y., Wu, X., Tong, X., Wang, X., Chang, Z., Mao, Y., et al. (2017). Circulating Tumor DNA Mutation Profiling by Targeted Next Generation Sequencing Provides Guidance for Personalized Treatments in Multiple Cancer Types. *Sci Rep* 7(1)**,** 583. doi: 10.1038/s41598-017-00520-1.

Yang, Z., Yang, N., Ou, Q., Xiang, Y., Jiang, T., Wu, X., et al. (2018). Investigating Novel Resistance Mechanisms to Third-Generation EGFR Tyrosine Kinase Inhibitor Osimertinib in Non-Small Cell Lung Cancer Patients. *Clin Cancer Res* 24(13)**,** 3097-3107. doi: 10.1158/1078-0432.CCR-17-2310.
